# Anger provocation in violent offenders leads to emotion dysregulation

**DOI:** 10.1038/s41598-017-03870-y

**Published:** 2017-06-15

**Authors:** Franca Tonnaer, Nicolette Siep, Linda van Zutphen, Arnoud Arntz, Maaike Cima

**Affiliations:** 1 Department of Research, Forensic Psychiatric Centre de Rooyse Wissel, Oostrum, The Netherlands; 2 Department of Clinical Psychological Science, Maastricht University, Maastricht, The Netherlands; 3 Department of Clinical Psychology, University of Amsterdam, Amsterdam, The Netherlands; 4 Department of Developmental Psychopathology, Radboud University, Nijmegen, The Netherlands; 5 Department of Research, Conrisq group, Zetten, The Netherlands

## Abstract

Anger and anger regulation problems that result in aggressive behaviour pose a serious problem for society. In this study we investigated differences in brain responses during anger provocation or anger engagement, as well as anger regulation or distraction from anger, and compared 16 male violent offenders to 18 non-offender controls. During an fMRI adapted provocation and regulation task participants were presented with angry, happy and neutral scenarios. Prior research on violent offenders indicates that a combination of increased limbic activity (involved in emotion), along with decreased prefrontal activity (involved in emotion regulation), is associated with reactive aggression. We found increased ventrolateral prefrontal activity during anger engagement in violent offenders, while decreased dorsolateral and ventrolateral prefrontal activity was found during anger distraction. This activity pattern was specific for anger. We found no exclusive pattern for happiness. In violent offenders, this suggests an increased need to regulate specifically during anger engagement and regulation difficulties when explicitly instructed to distract. The constant effort required for violent offenders to regulate anger might exhaust the necessary cognitive resources, resulting in a risk for self-control failure. Consequently, continuous provocation might ultimately contribute to reactive aggression.

## Introduction

Although anger is one of the most basic emotions, it is still widely misunderstood and ignored^1^. Elevated anger and inability to regulate anger are related to problematic and destructive conduct, including aggressive and violent behaviour, and therefore constitute a large burden to society^2, 3^. Violent offenders are characterized by extreme aggressive behaviour^4^, with anger as a risk factor for violent recidivism^5^.

While previous cognitive research using brain imaging techniques focused on punishment^6–8^, frustration^2^, or perceived threat as a trigger of anger or aggression^2^, emotion research has focused on the recognition of anger as an indicator for dysfunctions in experience and perception of anger^9, 10^. For instance, cognitive research using laboratory measurements tested the willingness to punish an opponent by giving electric shocks as assessment of aggression^6–8^. Furthermore, anger was operationalized by means of frustration after the lack of expected reward, ultimately resulting in anger^2^. Others confronted participants with a threatening environment or situation, with reactive aggression as a result^2^.

In emotion research anger is for instance operationalized in brain response differences during the perception of violent versus neutral images^11^. Another commonly used approach is assessing brain response differences during the perception of facial expressions contrasting neutral expressions with emotional ones, such as angry^12^, happy, fearful and sad^13^. Moreover, avoidance-approach paradigms have been utilized measuring the automatic action tendency responses to facial expressions while participants were instructed to avoid angry and approach happy faces^14^. Consequently, it is expected that deficits in emotion recognition within these emotion paradigms, ultimately lead to aggressive and violent behaviour^3^.

In other words, earlier studies using fMRI in violent offenders focused on punishment^6, 7^, frustration^2^, recognition of anger and automatic action tendency responses to facial expressions^14^. Up until now no fMRI study actually investigated anger engagement as well as anger distraction within a group of violent offenders (VOF) exhibiting anger problems, comparing them to non-offender controls (NOC).

Contributing to the theoretical understanding of the mechanisms underlying the regulation of anger, the present study is unique because of the comparison of violent offenders and non-offender controls during anger engagement and distraction. Nevertheless, in order to study specific effects of anger, one needs to know regulation responses to other emotions. The difference between anger engagement as opposed to a happy state^15^ in violent offenders is not well examined.

In this study we aimed to investigate engagement versus distraction of anger as well as happiness in the participants while measuring brain responses. To achieve this goal, we presented participants with audiotaped (anger, neutral and happy) stories, each within an *Engagement* condition instructing participants to focus on one’s emotional feeling, and a *Distraction* condition instructing participants to distract themselves from the presented stories during fMRI scanning. The current paradigm has demonstrated to provoke anger in a violent offender population^16^, but the paradigm has never been utilised in fMRI research before.

Research in violent offender populations, linking the reactive aggression to specific brain regions, suggests that a combination of decreased prefrontal activity along with increased limbic activity (e.g., amygdala) is related to antisocial behaviour and reactive aggression^2^. Following previous research of emotion regulation in non-patients and in violent offender populations, we hypothesized that the violent offenders displayed a neural network involved in emotion regulation, containing increased limbic activity, more specific in the amygdala, along with decreased prefrontal activity including the ventromedial, ventrolateral and dorsolateral prefrontal cortices^2, 17^.

## Results

### Manipulation check

The success of provocation and regulation was measured by analyzing the subjective rating of experienced emotional state by means of a visual analogue scale (VAS; 0 = *very happy* and, 100 = *very angry*) during scanning. The condition (engagement vs. distraction) x valence (anger, happiness vs. neutral) interaction was significant (*F*(1.34,44.19) = 11.42, *p* = 0.001, Greenhouse-Geisser). In line with our assumption pairwise comparisons showed that participants reported to be more angry during the engagement compared to the distraction condition (*t*(33) = −3.10, *p* = 0.004). This effect was also present for the happy stories (*t*(33) = 3.23, *p* = 0.002), with participants reported to be more happy during the engagement compared to the distraction condition. The neutral stories elicited no difference in responding between the engagement compared to the distraction condition (*t*(33) = −1.31, *p* = 0.199). Ratings per valence for each condition indicated group differences only in the subjective experience of anger, with the violent offenders (VOF) reporting more anger during both the engagement (*t*(32) = 2.14, *p* = 0.040) as well as the distraction condition (*t*(32) = 2.54, *p* = 0.016) compared to the non-offender controls (NOC). Moreover, in the self-evaluation of task success, VOF reported more difficulties with the engagement condition and believed to be less successful in focusing on their emotions compared to the NOC (Supplementary Table S1). No group differences were found regarding difficulties and success of the distraction condition.

### fMRI analyses

We investigated group differences in brain activity during anger (and happy) minus neutral^18^ engagement and distraction^19^ by means of four whole brain Random Effects (RFX) ANOVA’s, thresholded at *p* *<* 0.01 and corrected for multiple comparisons with a cluster size threshold (see Supplementary Materials for a more detailed description for fMRI data acquisition and preprocessing details).

### fMRI analyses during emotion provocation

**Figure 1:**
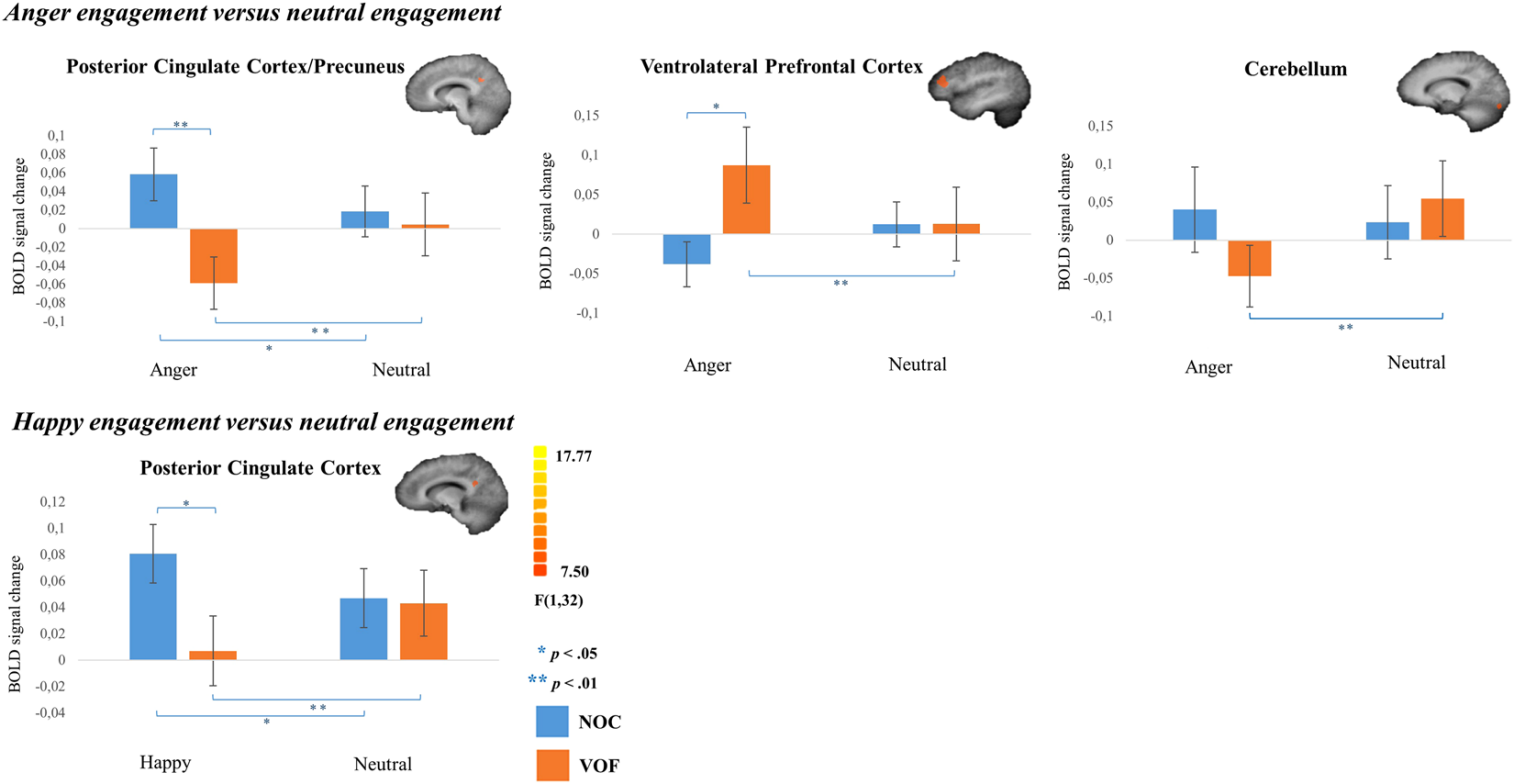
Cluster results on emotion provocation. Bar plots of beta values (+/− SEM) of clusters resulting from whole brain RFX ANOVA testing differences during engagement of anger and happy versus neutral stories.

The *F*-map of the provocation condition comprising anger stories [stimulus (*anger-engagement versus neutral-engagement*) x group (VOF versus NOC)] showed three clusters; the right posterior cingulate cortex (PCC)/Precuneus, left cerebellum and left ventrolateral prefrontal cortex (vlPFC) (Table 1). Happy provocation [stimulus (*happy-engagement versus neutral-engagement*) x group (VOF versus NOC)] showed significant activation of the right PCC (Table 1). Simple effects analyses of these resulting regions, indicated less PCC activity during anger as well as happy engagement in VOF, compared to NOC (Fig. 1; Table 1). Additionally, VOF exhibited a decreased activity during anger and happy engagement compared to neutral engagement, while NOC showed the opposite pattern. Concerning the cerebellum, in VOF, decreased activity during anger engagement versus neutral engagement was revealed (Fig. 1; Table 1). The vlPFC showed stronger activity for the VOF during anger versus neutral engagement compared to the NOC (Fig. 1; Table 1).

**Table 1:** Resulting clusters for RFX ANOVA testing differences between violent offenders (VOF) and non-offender controls (NOC).

Region	L/R	BA	Voxels (mm^3^)	Peak voxel, Talairach			F	*p*	Pairwise Comparisons		
				x	y	z			*t*	*p*	
*Anger Engagement versus Neutral Engagement*									*VOF vs NOC*		
Posterior cingulate cortex/Precuneus	R	31	499	11	−53	30	15.56	<0.001	−2.91	0.007	*Anger Engagement*
Cerebellum	L		472	−16	−83	−18	12.17	0.001	1.24	0.225	*Anger Engagement*
Middle frontal gyrus, ventrolateral	L	46	1023	−46	31	21	13.86	0.001	2.31	0.027	*Anger Engagement*
*Happy Engagement versus Neutral Engagement*											
Posterior cingulate gyrus	R	31	443	8	−50	27		<0.001	−2.15	0.039	*Happy Engagement*
*Anger Distraction versus Anger Engagement*									*Distraction vs Engagement*		
Posterior cingulate gyrus	L	30	1324	−16	−53	15	25.06	<0.001	−5.07	<0.001	*VOF*
									1.36	0.191	*NOF*
Inferior frontal gyrus, dorsolateral	L	9	468	−40	7	18	16.74	<0.001	−3.15	0.007	*VOF*
									2.60	0.019	*NOC*
Inferior frontal gyrus, ventrolateral	L	46	825	−40	37	0	17.24	<0.001	−2.55	0.022	*VOF*
									3.35	0.004	*NOC*
*Happy Distraction versus Happy Engagement*											
Posterior cingulate gyrus	L	30	763	−16	−53	15	17.37	<0.001	−5.37	<0.001	*VOF*
									−0.24	0.812	*NOC*
Inferior frontal gyrus, dorsolateral	L	9	958	−43	4	24	21.11	<0.001	−2.77	0.014	*VOF*
									3.76	0.002	*NOC*

Taken together, the vlPFC was exclusively active during anger engagement, whereas the PCC was active during anger and happiness engagement. All resulting brain regions were checked for confounding effects of medication within the VOF. No significant stimulus x medication interaction effect was found for any of the clusters.

### fMRI analyses during emotion regulation

**Figure 2:**
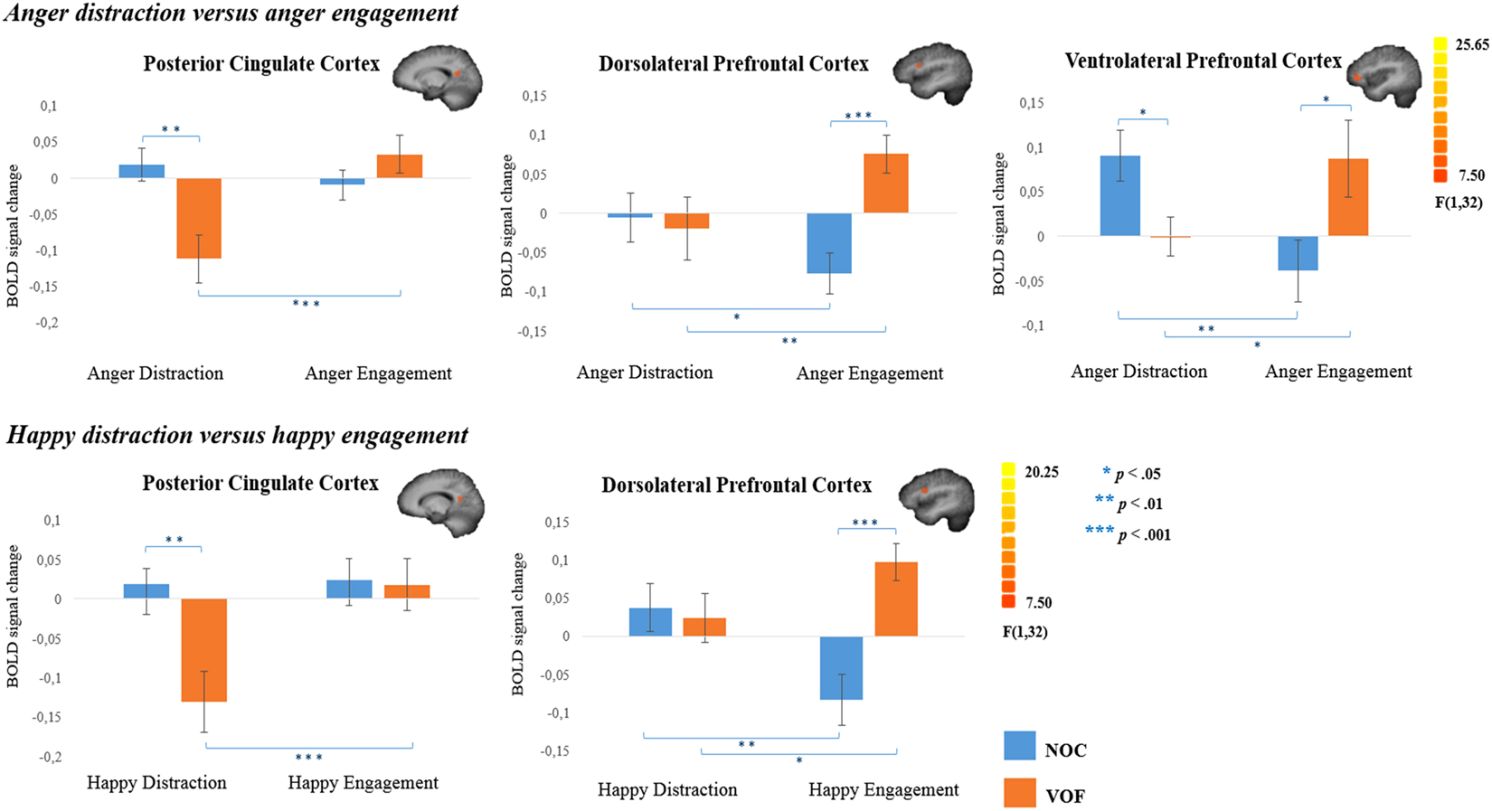
Cluster results on emotion regulation. Bar plots of beta values (+/− SEM) of clusters resulting from whole brain RFX ANOVA testing differences in distraction versus engagement in anger or happy stories.

The *F*-map of anger regulation [condition (*anger-distraction versus anger-engagement*) x group (VOF versus NOC)], showed three brain regions; the left PCC, left dorsolateral prefrontal cortex (dlPFC), and left vlPFC (Table 1). With respect to regulation of happy stories [condition (*happy-distraction versus happy-engagement*) x group (VOF versus NOC)], also showed significant activation in the left PCC, and left dlPFC (Table 1). Simple effects analyses of these resulting regions showed a decreased activity for distraction versus engagement during anger as well as happy stories in the PCC (Fig. 2; Table 1). In comparison to NOC, VOF revealed more activity in the dlPFC during the anger and happy engagement condition compared to the distraction condition. Furthermore, NOC showed decreased activity during distraction versus engagement in both angry and happy stories. Finally, VOF showed less activity when distracting from anger stories, and more activity during anger engagement than the NOC in the left vlPFC (Fig. 2; Table 1). Moreover, a decreased activity during distraction from anger versus engagement in anger was observed in VOF in the vlPFC, while NOC showed the opposite pattern with increased activity during anger distraction versus anger engagement.

**Figure 3:**
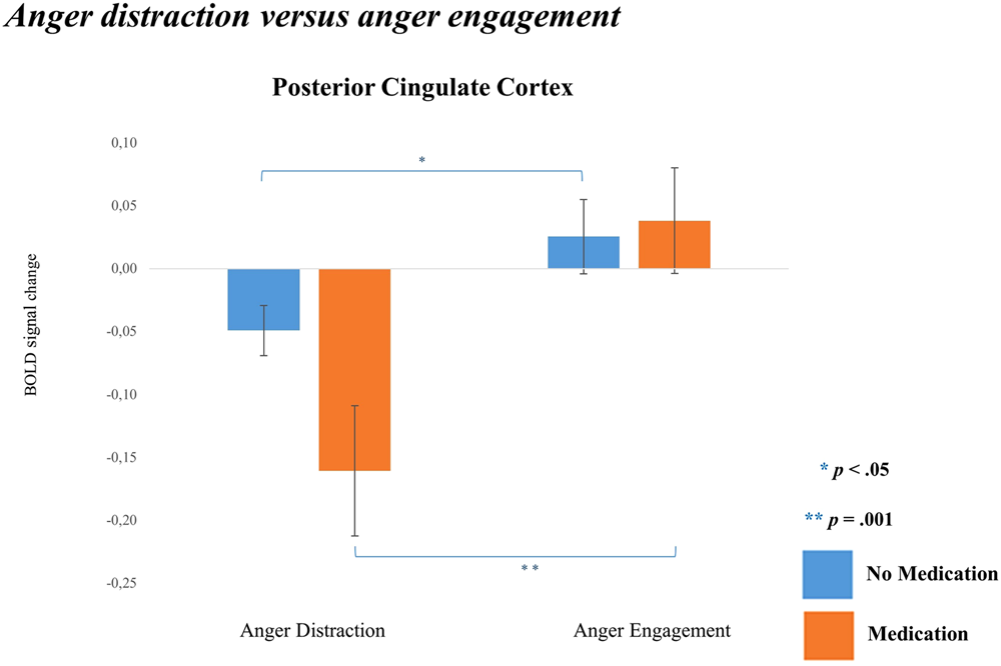
Bar plots of beta values (+/− SEM) of Posterior Cingulate Cortex activity resulting from simple effects analyses testing medication differences in distraction versus engagement during anger stories in the violent offender sample only.

Briefly, for all participants the vlPFC was exclusively active in the anger contrast of distraction versus engagement, while the PCC was again active in both emotional stories, along with the dlPFC. All resulting brain regions were checked for medication effects within the VOF. Only during anger stories the PCC showed a significant condition x medication interaction within VOF (*F*(1,14) = 6.32, *p* = 0.025), therefore these results should be interpreted with caution. Simple effects showed no main effect of medication. Moreover, both groups showed significantly less PCC activity during anger distraction compared to anger engagement, though the medication group showed a stronger difference between the two conditions (see Fig. 3). For this reason, the effect of medication in the PCC during anger distraction appears marginal.

### fMRI analyses focussed on the amygdala

**Figure 4:**
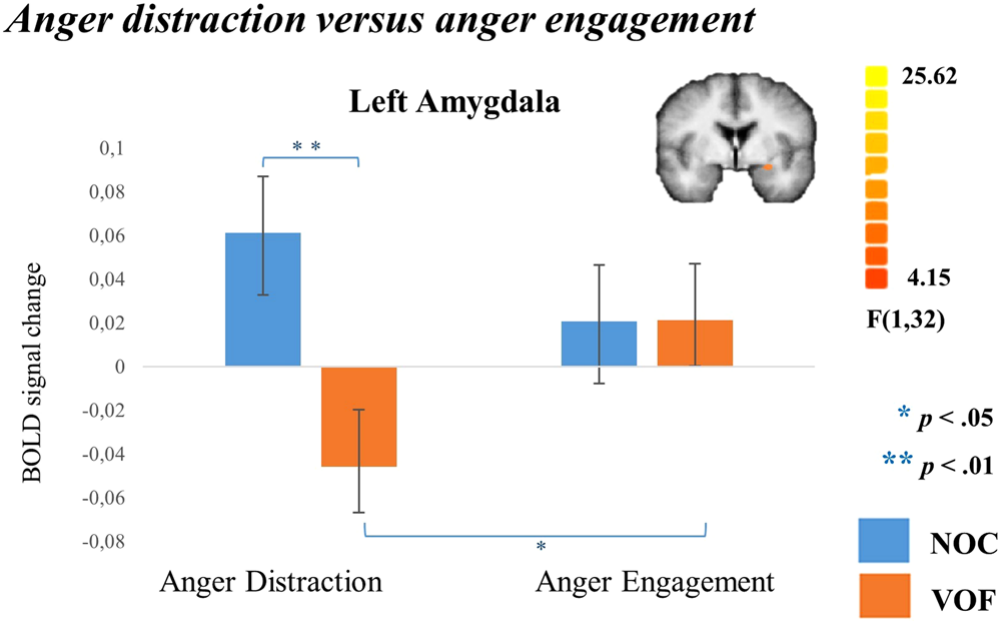
Amygdala results. Bar plots of beta values (+/− SEM) of Amygdala activity resulting from whole brain RFX ANOVA at a more lenient significance level, testing differences in distraction versus engagement during anger stories.

Research in violent offender populations suggests amygdala hyperactivity in the brain network related to reactive aggression^2, 20^. In the whole brain analyses we did not find a differential activity in the amygdala. However, the amygdala is particularly sensitive for artifacts during scanning due to its location next to air-filled spaces. This may lead to null findings reflecting rather signal loss than absence of brain activity^21^. Therefore, we additionally examined whole brain RFX amygdala activity at a more lenient significance level. In the left amygdala VOF showed, relative to NOC (*t*(32) = 2.93, *p* = 0.006), a decreased activity during anger distraction compared to anger engagement (*t*(15) = −2.359, *p* = 0.032; Fig. 4). VOF showed less amygdala activity during distraction from anger compared to NOC (*t*(32) = 2.925, *p* = 0.006; Fig. 4), while no group differences were found during anger engagement (*t*(30.36) = −0.012, *p* = 0.991).

### Correlation Aggressiveness, VLPFC and Amygdala

Additional analysis on the correlation between the brain activity in the VLPFC and the amygdala on one hand and data on self-reported aggressiveness (Aggression Questionnaire (AQ^22^) & Reactive-Proactive Questionnaire (RPQ^23^)) on the other hand, revealed that less activity in the amygdala during anger regulation is related to aggression assessed with the RPQ (*r*(32) = *−*0.45, *p* = 0.007 for RPQ-Total Score; *r*(32) = *−*0.41, *p* = 0.018 for the RPQ-Reactive Scale; *r*(32) = *−*0.45, *p* = 0.008 for the RPQ-Proactive Scale). And less activity in the VLPFC during anger regulation is related to aggression assessed with both the RPQ (*r*(32) = *−*0.47, *p* = 0.005 for RPQ-Total Score; *r*(32) = *−*0.44, *p* = 0.01 for the RPQ-Reactive Scale; *r*(32) = *−*0.44, *p* = 0.01 for the RPQ-Proactive Scale) and the AQ (*r*(32) = *−*0.45, *p* = 0.005 for AQ-Total Score before the task paradigm and *r*(30) = *−*0.45, *p* = 0.01 for AQ-Total Score after the task paradigm).

## Discussion

The aim of the current study was to examine the brain responses during an anger engagement and distraction task^18, 19^ in violent offenders compared to non-offender controls. In contrast to our expectations violent offenders showed higher activity in the vlPFC and less activity in the PCC during anger engagement compared to non-offender controls. The results of the distraction condition showed decreased activity in the PCC, dlPFC and vlPFC when violent offenders were instructed to regulate, or distract themselves during anger stories, whereas non-offender controls showed an increase activity in dlPFC and vlPFC. However, the effects in the PCC and dlPFC were not specific for anger, as similar responses were shown for happy stories. Activity in the vlPFC during engagement and distraction was specific for anger stories. And, less activity in the VLPFC during anger distraction was related to self-reported aggression (both RPQ^23^ and the AQ^22^) indicating a potential link between aggression and decreased VLPFC activity.

Amygdala activity was only found at a liberal level of significance, showing decreased activity in violent offenders during distraction from anger compared to anger engagement. Moreover, no group differences were found during anger engagement, while during anger distraction the violent offenders showed less amygdala activity compared to the non-offender controls, indicating less amygdala sensitivity during anger distraction. Additional, less activity in the amygdala during anger distraction was related to self-reported aggression (RPQ^23^) indicating a potential link between aggression and decreased amygdala activity.

Kohn and colleagues^24^ suggest that the vlPFC is a relay station of information towards the dlPFC and selects and initiates reappraisals, while they marked the dlPFC as the central regulatory brain area, engaged in attending to and maintaining reappraisal in working memory. According to this framework, less activity in the vlPFC for the violent offenders specific during anger distraction might indicate a dysfunction in signaling the need to regulate. Though initiation to regulate is primary expected in the distraction condition as shown in the non-offender controls, violent offenders already showed overactive initiation to regulate emotions during the anger engagement instruction. This constant initiated reactivity, specific for anger stories, might exhaust cognitive sources required to regulate or distract from anger in violent offenders. Furthermore, the dlPFC is associated with regulatory and inhibitory processes of cognitive control^15, 17^. The reduced dlPFC activity shown in the violent offenders during anger as well as happy distraction might indicate a general top-down problem resulting in impaired cognitive control of emotions. This is in line with previous research in antisocial and aggressive behavior^25, 26^. Remarkable, self-report after scanning did not indicate any regulation difficulties within violent offenders, suggesting not being attentive of these potential difficulties indicated by overactive initiation to regulate.

We had no specific expectations about the PCC as regulation theories and earlier research in offenders did not point to this brain area as specific for anger (or emotion) distraction. The PCC has been associated with self-referential processing and recall of autobiographic emotional events^27–30^. It could be speculated that the decreased activity of the PCC found in the violent offenders during anger and happy engagement indicate that violent offenders interpret, or recall, the emotional stories less in relation to themselves. Moreover, earlier research in offender populations showed that offenders characterized with emotional detachment and antisocial behavior were more impaired in the recall of emotional material than healthy control males^31^. The current results might indicate less emotional regulation during affective processing in violent offenders.

Though strengths of the current study are the direct comparison of violent offenders with non-offender controls, the naturalistic provocation, and the use of not only anger and neutral, but also happy stories, a number of limitations should also be acknowledged. First, as previously described we only tested men, which limits the generalizability of our results.

Second, the sample size was small, thus results should be interpreted with caution. Although it is challenging to collect data in forensic samples future studies should increase the statistical power by increasing the sample size and examine more subtle effects. Third, the generalizability of the forensic sample from a high security hospital might be limited due to the fact that all offenders received a treatment program including anger management. Perhaps, adding a non-care (e.g. penitentiary) forensic sample in future research could provide more insight. Fourth, some offenders acquired prescribed medication for clinical reasons. As medication is known to affect the neural brain responses^32^, this might have influenced our results. Therefore, pairwise comparisons were performed within the violent offenders to check for any possible relation between medication intakes. With the exception of the PCC during anger distraction, interactions of medication within the clusters were not significant, suggesting medication did not influence these results. Further, pairwise comparisons of the PCC showed that although the medication x condition interaction was significant, both the medicated and the non-medicated group showed a significant pattern of reduced PCC activation during anger distraction compared to anger engagement, though this effect was stronger in the medicated group. Thus, it seems unlikely that medication caused the effects found in the PCC during anger distraction. It seems more likely that violent offenders with a stronger reduction in PCC activation during anger distraction are more likely to get medication, further suggesting that reduced PCC activation plays a role in causing problems in these offenders. Fifth, we cannot rule out interference of distracting themselves during the task, and unequal task engagement, even though participants were explicitly instructed not to do so. Alternatively, less activity in the vlPFC for the violent offenders specific during anger distraction might indicate difficulties in distracting themselves from the stories, instead of a dysfunction in signaling the need to regulate. However, we explicitly used emotional (anger and happy) versus neutral story contrasts with equal instructions and equal length to rule out the effect of story processing. Further, all participants were asked to review the difficulty and success of the engagement as well as the distraction condition and no group differences were found regarding difficulties and success of the distraction condition. Moreover, interference during the experimental task seems unlikely since participants reported focusing on emotion (e.g. imagination) in the engagement condition and using regulation strategies (like thinking about other situations, counting etc.) during distraction in self-report after scanning, as well as in the manipulation check of the subjective rating of experienced emotion during scanning.

In conclusion, our results suggest increased initiation to regulate as indicated by increased vlPFC activity during anger engagement and less during anger distraction in violent offenders. Additionally, when explicitly instructed to regulate by means of distraction, results hint to general emotion regulation impairments in violent offenders. The constant effort for regulation in violent offenders might exhaust cognitive sources required to regulate anger in violent offenders, resulting in a risk factor for self-control failure^33^. Ultimately this might contribute to reactive aggression when continuously provoked. Consequently, future research should investigate whether this decreased dlPFC during anger distraction is a result of vlPFC dysfunction, or signals a broader regulation problem. Moreover, more insight in the effects of dysregulation and possible regulatory exhaustion risk to regulate anger in violent offenders is needed. A recent study showed that failure of self-control may have little to do with depleted resources^34^, therefore future research should test the possible explanation of exhaustion and should be replicated in extent.

## Methods

### Participants

A total of 34 males participated in the study. The violent offenders (VOF; *n* = 16) were convicted for a violent crime (e.g. (attempted) manslaughter or murder, property crime with violence, bodily harm, domestic violence), and recruited from a male population at the Forensic Psychiatric Centre *de Rooyse Wissel* and its ambulatory care at *De Horst*. The non-offender control group (NOC; *n* = 18), were recruited from a participant database and consisted of male participants with no history of violent behavior, matched on age, education level, and dominant handedness with the VOF (see Table 2).

**Table 2:** Demographic and clinical characteristics of the sample and group differences for behavioral anger measurements using Independent-Samples t-Tests.

	Violent offenders		Non-offender controls		Statistics	
	*M/n*	*SD/%*	*M/n*	*SD/%*	*t/χ* ^2^	*p*
Age, M SD	35.81	7.17	34.39	13.37	0.39	0.70
*Education*, *n %*						
Secondary school	16	88.9	14	87.5	−0.12	0.90
College	2	11.1	2	12.5	0.12	0.90
*Disorder*, *n %*						
Substance dependence	13	81.3	0	—	8.06	0.001
Depressive episode past	5	33.3	1	5.6	1.95	0.07
PTSS	8	50.0	0	—	3.87	0.002
Antisocial PD	9	56.3	0	—	4.39	0.001
Borderline PD	3	18.8	0	—	1.86	0.08
Other PD	4	25.0	0	—	2.24	0.04
*General Anger*, *M SD*						
RPQ-Total	21.9	8.7	6.0	4.3	6.7	<0.001
RPQ-Reactive	14.4	5.3	5.0	3.4	6.0	<0.001
RPQ-Proactive	7.6	4.7	1.0	1.6	5.3	<0.001
*Anger Pre Scanning*, *M SD*						
AQ-Total	51.8	18.3	28.3	13.1	4.3	<0.001
Anger-STIAT	781.6	280.2	767.8	166.4	.30	0.77
*Anger Post Scanning*, *M SD*						
AQ-Total	53.4	16.1	27.4	14.3	4.8	<0.001
Anger-STIAT	679.3	152.0	719.9	138.6	1.6	0.12

*Note*. PTSS = posttraumatic stress disorder; PD = personality disorder; RPQ = Reactive-Proactive Questionnaire, AQ = Aggression Questionnaire, Anger-STIAT = Anger-Single Target Implicit Association Test.

Participants were screened for MRI contraindications and were excluded if they were younger than 18 years, had an IQ below 80, or reported psychotic symptoms. All participants were Dutch, and ranged in age from 20 to 58 years (*M* = 35.1, *SD* = 10.77). As to their educational level, 88.2% had attended secondary school and 11.8% had attended college. Exclusion criteria for the non-offender controls included current DSM-V psychiatric disorders. Demographic and clinical characteristics of the sample are summarized in Table 2.

Psychopathy Checklist-Revised (PCL-R^35, 36^ data were collected for a subsample of *n* = 10 VOF of which data was available: total scores ranged from 12 to 28 (*M* = 21.4, *SD* = 6.2, with a score of 26 or above indicating psychopathy^37, 38^. Internal consistency in the current sample was excellent (Cronbach’s alpha = 0.83 for PCL-R total score). Pathology was scored for scientific purpose by semi-structured interviews^39, 40^ based on the Diagnostic and Statistical Manual of Mental Disorders (DSM-IV^41^. All interviews were scored by forensic psychologists who were trained to administer the interview, resulting in a consensus score arrived through discussion of scoring differences.

In line with our assumption that VOF showed a higher general level of anger compared to the NOC (measured with the Aggression Questionnaire^23^) and a tendency towards higher reactive aggression scores (measures with the Reactive-Proactive Questionnaire^24^; *t*(32) = −6.0, *p* *<* 0.001). Furthermore, results show faster response time in the post Anger-Single Target Implicit Association Test^42^, indicating a stronger ‘self’-‘anger’ concept association after provocation, but these results are non-significant and there is no differences in implicit anger between VOF and NOC (see Table 2).

### Ethics

The Ethical Committee of Maastricht University and the Research Committee of de Rooyse Wissel approved the methods and procedures described in the research protocol, all of which were performed in accordance with the Declaration of Helsinki. They received written and oral instruction emphasizing that participation was not related to treatment or prospects for release, and that participants were free to withdraw from the study at any time. After description of the study, written informed consent was obtained from each subject in accordance with de Rooyse Wissel, its ambulatory care De Horst and Maastricht University. Moreover, participants received a financial compensation for their participation. Originally 45 participants were recruited. However, five participants withdraw due to a lack of interest (two VOF, and three NOC), one VOF did not enter the scanner because of fMRI contraindications (history of epilepsy), data of four participants (two VOF, and two NOC) was never processed due to technical problems during scanning (e.g. malfunction of the joystick, of audio or incomplete functional coverage), and one control participant was recruited purely to pilot the test setting.

### Procedure

Participants first completed the Reactive-Proactive Questionnaire (RPQ^23^), the Aggression Questionnaire (AQ^22^) and the Anger-Single Target Implicit Association Test (Anger-STIAT^43^) in randomized order to determine the general anger level and possible preference towards specific types of aggression. In order to familiarize with the study paradigm and to check the auditory comprehension, participants started with a practice session of the emotion paradigm in the MRI (including active task engagement by means of subjective VAS state-emotional ratings after each audio fragment). To measure provocation and regulation of anger and happiness an adapted version of the Articulated Thoughts in Simulated Situations paradigm (ATSS) for fMRI^43^ was used. In the ATSS paradigm participants either were provoked or needed to attend and respond naturally, or they were instructed to regulate their emotional state by distracting themselves from, six narrative stories differing in their affective content (happy, anger, and neutral). In order to capture the subjective rate of experienced emotion a visual analogue scale (VAS) was used. As part of the scanning session participants also underwent two resting state scans (data presented separately^44^. After the scan session, all participants again completed the AQ^22^ and the Anger-STIAT^43^ to determine the post anger level, followed by an Exit-Questionnaire evaluating scanner and task experience (Supplementary Table S1).

### Experimental task

In the current study an adapted MRI version of the Anger Articulated Thoughts during Simulated Situations (ATSS) paradigm^45^ was utilized in order to elicit anger and happy provocation and regulation. The ATSS is a cognitive assessment of audio taped thoughts and beliefs, in which subjects are asked to imagine and respond to audiotape presented situations. In the fMRI version of the ATSS^45^ participants were presented with two ATSS conditions: an *Engagement* condition and a *Distraction* condition. During the Engagement condition participants were instructed to focus on their emotional feelings during the presented audio situations. The Distraction condition required participants to regulate their emotional state by distracting themselves from the presented audio situations. Both conditions included three different situations; a happy, neutral, and an anger situation^45^. The presentations of the conditions were counterbalanced and the order of the audio taped situation was randomized per participant. Each situation was divided into seven segments of 15 to 20 seconds. At the end of each segment there was a tone, followed by a silence of 15 seconds. The silence was followed by a visual analogue scale (9 seconds), which participants used to rate their emotional state at that moment (0 = *very angry* and, 100 = *very happy*). Previous research shows the ATSS is able to induce anger in a violent offender population^16^ and is related to specific anger cognition biases in inmate partner abusers^46^. Additional assessments included the RPQ^23^, the AQ^22^, the Anger-STIAT^43^ and an Exit-Questionnaire. See Supplementary Materials for the measure descriptions other than the fMRI paradigm.

### Statistical analysis

Because each condition (engagement and distraction) was recorded in a separate run, condition was dummy coded. Therefore, the applied general linear model included 10 predictors: anger-engagement, anger-distraction, happy-engagement, happy-distraction, neutral-engagement, neutral-distraction, sound fragments during engagement, sound fragments during distraction, visual analogue scale rating during engagement and visual analogue scale rating during distraction. Moreover, white matter and ventricle reference time courses were created for each participant and added to the general linear model (GLM) along with the six motion correction parameters. The following four whole brain random effect (RFX) ANOVA were carried out (the first two to measure engagement and the latter two to measure distraction): first, to investigate anger engagement: stimulus (*anger-engagement vs*. *neutral-engagement*) x group (VOF vs. NOC); second, to investigate happy engagement: stimulus (*happy-engagement vs*. *neutral-engagement*) x group (VOF vs. NOC); third, to examine anger distraction contrasting the engagement to the distraction condition within the anger valence: stimulus (*anger-distraction vs*. *anger-engagement*) x group (VOF vs. NOC), and fourth, to examine happy distraction contrasting the engagement to the distraction condition within the happy valence: stimulus (*happy-distraction vs*. *happy-engagement*) x group (VOF vs. NOC). The resulting F-maps were thresholded at significance level of *p* < 0.01, and cluster size being 16 for the anger maps and 15 for the happy maps. The minimal cluster size was determined with the cluster-level estimation plug-in in BrainVoyager, which implements a Monte Carlo simulation for multiple comparisons correction of *p* < 0.05 (1000 simulation^47^. For detailed analyses of the resulting clusters beta values of each predictor per participant were exported to SPSS version 22 (IBM Corporation, New York). When Mauchly Test of Sphericity was affected Greenhouse-Geisser correction was applied. Additionally, all resulting brain regions were checked for medication effects by means of pairwise comparisons of all contrasts (emotion engagement and emotion distraction) x medication interaction in VOF only (see Fig. 3). Medication was only prescribed for clinical reasons within the VOF (*n* = 9, within the VOF sample of *n* = 16, all psychotropic medication, mostly antidepressants), and no psychotropic medication was reported in the non-offender controls. The relationship between self-reported aggressiveness (AQ^22^, RPQ^23^) and brain activity in the VLPFC as well as the amygdala assessed with correlational analyses.

### Data availability

The datasets generated during and/or analyzed during the current study are available from the corresponding author on reasonable request.

## Electronic supplementary material

Supplementary Materials
