# Supplementary material for: Anger provocation in violent offenders leads to emotion dysregulation
Source: Sci Rep. 2017 Jun 15;7:3583. doi: 10.1038/s41598-017-03870-y (PMC5472615; doi:10.1038/s41598-017-03870-y)
Supplement: Supplementary file 1 — Supplementary Materials [file 41598_2017_3870_MOESM1_ESM.doc]

**Supplementary Materials**

**Anger provocation in violent offenders leads to emotion dysregulation**

Authors and affiliations:

Franca Tonnaer*^1,2^, Nicolette Siep^2^, Linda van Zutphen^2^, Arnoud Arntz^2,3^, and Maaike Cima^4,5^

^1^ Department of Research, Forensic Psychiatric Centre de Rooyse Wissel, The Netherlands.

^2^ Department of Clinical Psychological Science, Maastricht University, The Netherlands.

^3^ Department of Clinical Psychology, University of Amsterdam, The Netherlands.

^4^ Department of Developmental Psychopathology, Radboud University, The Netherlands.

^5^ Department of Research, Conrisq group, The Netherlands.

**Methods**

**Measures**

**Reactive-Proactive Questionnaire (RPQ[[1]](#endnote-2)).** The RPQ was used as a self-report of aggression during life time. The RPQ consists of 23 items that are rated on a 3-point Likert-scale (0 = *never* and 2 = *always*). The questionnaire includes two subscales of aggression: the proactive subscale, which measures proactive aggression in items such as ‘How often have you used force to get others so what you want?’, and the reactive subscale measuring reactive aggression in items such as ‘How often have you got angry or mad or hit others when teased?’. Scores of the subscales are calculated by summing the values for the items. Research has shown good internal reliabilities for total RPQ, and reactive and proactive subscale scores with all reliability coefficients exceeding .81^1^,[[2]](#endnote-3). The RPQ demonstrated good construct validity, convergent validity, criterion validity, and discriminant validity^1^. Internal consistency in the current sample was excellent (Cronbach’s alpha = .95 for RPQ total score, .91, for the proactive subscale, and .95, for the reactive subscale).

**The Aggression Questionnaire (AQ[[3]](#endnote-4)).** The AQ is a self-report of 29 items, each of which is evaluated on a 5-point Likert scale (0 = *definitely disagree* and 4 = *definitely agree*). Scores of two items are recoded reversed and the total score (29 items) and subscales are calculated by summing the values for the items. The AQ contains a four factor structure, presented in according subscales denoting; 1) Physical Aggression (9 items such as ‘Once in a while I can’t control the urge to strike another person.’), 2) Verbal Aggression (5 items such as ‘I can’t help getting into arguments when people disagree with me.’), 3) Hostility (8 items such as ‘When people are especially nice, I wonder what they want.’) and 4) Anger (7 items such as ‘I sometimes feel like a powder keg ready to explode.’). The AQ has a test-retest reliability, ranging between .72 and .80^3^,[[4]](#endnote-5) and good construct validity^3^. Internal consistency in the current sample was excellent (Cronbach’s alpha = .92 for AQ total score before scanning and .93 for AQ total score after scanning).

**Anger-Single Target Implicit Association Test (Anger-STIAT[[5]](#endnote-6)).** The STIAT is a single target variant of the Implicit Association Test (IAT[[6]](#endnote-7)). The STIAT^6^ measures the extent to which a target concept (e.g., a cake) is associated with two attributes (e.g., pleasant and unpleasant). When highly associated categories share the same response key (e.g., flower-pleasant and insect-unpleasant) performance is fast and accurate. Conversely, when negative associated categories share a response key (e.g., flower-unpleasant and insect-pleasant) performance is slow and errors increase. For this Anger-STIAT, self-concept was the target category stated as ‘I’ and the two attribute categories were ‘anger’ and ‘peaceful’. In the congruent condition the same response key is assigned for both the target words (the participants first name, last name, date of birth, age, address) and the peaceful words (e.g., calm, relaxed, happy, pleasant, balanced). In the incongruent condition the target words and the anger words (angry, furious, rebellious, irascible) share the same response key. The STIAT effect is calculated by subtracting the reaction time for the incongruent condition from the congruent condition^6^,[[7]](#endnote-8). Responses were taken via the keyboard of the computer, comprising two response buttons (Q and P). Response keys were counterbalanced, as were the congruent and incongruent trials. Computer recorded all reaction times and errors. Words were presented on the center of the screen, blue in the target category and black in the attribute category, against a white background. Each word was presented on the screen and participants were required to respond as quickly as possible. Whenever the participant responded, the word disappeared and the next trial commenced later. The reliability and validity of the IAT has been proven in different social domains as racism[[8]](#endnote-9) and self-esteem[[9]](#endnote-10). The current Anger-STIAT has proven its validity in earlier research on aggression and the self-concept^5^. Internal consistency in the current sample was good before scanning (Cronbach’s alpha = .84) and poor after scanning (Cronbach’s alpha = .53).

**Exit-Questionnaire.** The exit-questionnaire consisted of various 100 mm visual analogue scales, assessing possible influential aspects as physical discomfort inside the scanner (0 = *no physical complaints* and*,* 100 = *a lot of physical complaints*), nervousness (0 = *very nervous* and, 100 = *not nervous at all*), disturbance by the scanner noise (0 = *no hinder* and, 100 = *a lot of hinder*) or concentration problems (0 = *could not concentrate* and, 100 = *could concentrate very well*). In addition, participants were asked about task difficulty (0 = *very difficult*, and 100 = *not difficult at all*) and success (0 = *not at all successful*, and 100 = *very successful*) in performing the emotion engagement and distraction task. Moreover, we asked all participants about the strategy they used to engage and to regulate their emotional state by distracting themselves from their emotions.

**fMRI data acquisition**

Data was collected using a Siemens Magnetom Allegra 3T head-only scanner equipped with a birdcage headcoil (Siemens Medical Systems, Erlangen, Germany). Participants were scanned in head first supine position. Head movements were minimalized using foam paddings. Functional data was acquired using a standard echo-planar imaging (EPI) sequence resulted from T2*-weighted functional measurements with a repetition time (TR) of 2000 ms, echo time (TE) 30 ms, flip angle = 90°, 435 volumes with 32 axial slices, 3 x 3 x 3 mm, field of view (FoV) 192 mm and matrix 64 x 64. The T2*-weighted slices were optimized with a negative tilt of 30°, to minimize susceptibility and distortion artifacts within the amygdala[[10]](#endnote-11). Anatomical data was collected using a high resolution T1-weighted gradient echo with the following parameters: TR = 2250 ms, TE = 26 ms, flip angle = 9°, 192 volumes in the sagittal plane, voxel dimensions 1 x 1 x 1 mm and FoV = 256 mm.

**fMRI data preprocessing**

FMRI data preprocessing and analyses were conducted with Brainvoyager QX v2.8 (Brain Innovation, Maastricht, The Netherlands). The first two volumes of each run were discarded due to saturation effects. Preprocessing included sinc interpolation slice time correction, mean intensity adjustment if necessary due to scanner noise (*n* = 7), high-pass GLM-Fourier temporal filtering with 2 sines/cosines cycles and motion correction with trilinear interpolation for 3D motion detection and sinc interpolation actual motion correction[[11]](#endnote-12). In all anatomical scans the tissue was peeled from the skull and corrected for intensity in homogeneities. Then, functional and anatomical data was coregistered per run and for each run a volume time course (VTC) was created, followed by spatial normalization using standard Talairach transformation and 6 mm spatial smoothing with a full-width-at-half-maximum isotropic Gaussian kernel.

Supplementary Table S1.

*Group differences in exit questionnaire using Independent-Samples t-Tests.*

|  |  | Violent Offenders | | Non-offender controls | | Statistics | |
| --- | --- | --- | --- | --- | --- | --- | --- |
|  |  | *M* | *SD* | *M* | *SD* | *t* | *p* |
| *General scanning comfort* | |  |  |  |  |  |  |
|  | Physical discomfort | 24.4 | 28.3 | 30.8 | 26.9 | -0.66 | .51 |
|  | Concentration | 75.3 | 21.2 | 74.1 | 21.8 | -0.15 | .88 |
|  | Nervousness | 68.3 | 35.5 | 85.2 | 15.2 | -1.62 | .12 |
|  | Disturbance scanner noise | 49.8 | 29.4 | 34.9 | 24.6 | -1.71 | .10 |
| *Task evaluation* | |  |  |  |  |  |  |
|  | Task apprehensibility | 75.9 | 22.6 | 69.8 | 30.0 | 0.66 | .52 |
|  | Task identifiability | 90.9 | 9.4 | 89.6 | 13.8 | 0.33 | .75 |
|  | Difficulty emotion engagement | 62.2 | 32.4 | 85.1 | 15.6 | -2.50 | .02 |
|  | Difficulty emotion distraction | 61.2 | 34.0 | 75.4 | 22.9 | -1.38 | .18 |
|  | Success emotion engagement | 67.7 | 21.2 | 83.6 | 12.1 | -2.58 | .02 |
|  | Success emotion distraction | 64.5 | 17.8 | 75.6 | 22.7 | -1.53 | .14 |

**References**

1. Raine, A. *et al.* The Reactive-Proactive Aggression Questionnaire: Differential correlates of reactive and proactive aggression in adolescent boys. *Aggress Behav* **32,** 159-171, doi: 10.1002/ab.20115 (2006). [↑](#endnote-ref-2)
2. Cima, M. J., Raine, A., Meesters, C. & Popma, A. Validation of the Dutch Reactive Proactive Questionnaire (RPQ): Differential correlates of reactive and proactive aggression from childhood to adulthood. *Aggress Behav* **39,** 99-113, doi: 10.1002/ab.21458 (2013). [↑](#endnote-ref-3)
3. Buss, A. H., & Perry, M. The Aggression Questionnaire. *J Pers Soc Psychol* **63,** 452-459, doi: 10.1037/0022-3514.63.3.452 (1992). [↑](#endnote-ref-4)
4. Hornsveld, R. H. J., Muris, P., Kraaimaat, F. W., & Meesters, C. The Aggression Questionnaire in Dutch violent forensic psychiatric patients and secondary vocational students. *Assessment* **16,** 181-192, doi: 10.1177/1073191108325894 (2009). [↑](#endnote-ref-5)
5. Lobbestael, J., Arntz, A., Cima, M., & Chakhssi, F. Effects of induced anger in patients with antisocial personality disorder. *Psychol Med* **39,** 557-568, doi: 10.1017/S0033291708005102 (2009). [↑](#endnote-ref-6)
6. Greenwald, A. G., McGhee, D. E., & Schwartz, J. K. L. Measuring individual differences in implicit cognition: The implicit association test. *J Pers Soc Psychol* **74,** 1464-1480 (1998). [↑](#endnote-ref-7)
7. Karpinski, A., & Hilton, J. L. Attitudes and the Implicit Association Test. *J Pers Soc Psychol* **81,** 774, doi: 10.1037//0022-3514.81.5.774 (2001). [↑](#endnote-ref-8)
8. Dasgupta, N., & Greenwald, A. G. On the malleability of automatic attitudes: Combating automatic prejudice with images of admired and disliked individuals. *J Pers Soc Psychol* **81,** 800–814, doi: 10.1037//0022-3514 81.5.800 (2001). [↑](#endnote-ref-9)
9. Greenwald, A. G., & Farnham, S. D. Using the implicit association test to measure self-esteem and self-concept. *J Pers Soc Psychol* **79,** 1022-1038, doi: 10.1037/KK122-3514.79.6.1022 (2000). [↑](#endnote-ref-10)
10. Morawetz, C. *et al.* Improved functional mapping of the human amygdala using a standard functional magnetic resonance imaging sequence with simple modifications. *Magn Reson Imaging***26,** 45-53, doi: 10.1016/j.mri.2007.04.014 (2008). [↑](#endnote-ref-11)
11. Goebel, R., Esposito, F., & Formisano, E. Analysis of functional image analysis contest FIAC) data with BrainVoyager QX: from single-subject to cortically aligned group general linear model analysis and self-organizing group independent component analysis. *Hum Brain Mapp* **27,** 392-401, doi: 10.1002/hbm.20249 (2006). [↑](#endnote-ref-12)
